# Evicted from paradise: Methodological challenges of posthuman healthcare research

**DOI:** 10.1177/13634593261442953

**Published:** 2026-04-21

**Authors:** David A. Nicholls, Clair Hebron, Shirley Chubb

**Affiliations:** 1 Auckland University of Technology, New Zealand; 2 University of Brighton, UK; 3 University of Chichester, UK

**Keywords:** vitalism, transcendence, substance, process, correlation

## Abstract

Interest in posthuman concepts and ideas, philosophies and theories has grown enormously over the last 25 years, and posthumanism is now one of the most vibrant and innovative frontiers in healthcare thinking. At its most basic, posthumanism is a philosophical approach that decentres the human and considers other non-human or more-than-human objects as equally important. But this description belies the many challenges posthumanism presents to the researcher. There are many competing approaches to consider, there is often opaque language to navigate, and there are many structural problems to overcome. In this paper we tackle three major methodological challenges: vitalism, or the question of what gives life to things; transcendence, and the substance problem; and correlation, or latent anthropocentrism. We consider how it might be possible to research with a process-based ontology in a world dominated by substance-based principles. And we conclude with four related recommendations: a focus on key principles, concept creation, deep reading and attention to ontological slippage, before reflecting on our own experiences researching walking for people living with persistent pain.

## Introduction

Interest in posthuman concepts and ideas, philosophies and theories has grown enormously over the last 25 years, and posthumanism is now one of the most vibrant and innovative frontiers in healthcare thinking (Aagaard, 2022; Denzin and Giardina, 2023: 2; Harris and Rousell, 2022; Jackson, 2017; Kuby and Bozalek, 2023; Kuntz and St.Pierre, 2021; Mazzei and Jackson, 2023; St. Pierre, 2023, 2024).

Built from a collective desire to critique and re-imagine the centripetal power that humanism has had over Western thinking and practice since the Renaissance, posthumanism draws from a wide range of approaches including schema as diverse as pre-Socratic metaphysics, vitalism, C18 romanticism, Indigenous cosmologies, monadism, Hinduism, Buddhism and a range of other spiritual traditions, process philosophy, intersectional feminism, quantum physics and cybernetics. In doing so it has given birth to a diverse set of schools and approaches, including critical feminist new materialism, actor-network theory, assemblage theory, consciousness studies, ecocriticism and posthuman ethics, posthuman healthcare informatics, object-oriented ontology and speculative realism, postcolonial and indigenous studies, post-critical disability studies, process theology, speculative pragmatism, systems theory and theories of non-human agency. These approaches have also generated a host of neologisms that encompass the posthuman, the post-anthropocentric non-human, the inhuman, the new human, multi-species entities and posthuman personhood.

This complexity would seem to contradict Adam, Juergensen and Mallette’s assertion that posthumanism is, at its simplest, a ‘philosophical approach that decentres the human and considers other non-human or more-than-human objects as equally important’ (Adam et al., 2021). Thinking that new forms of research might be this straightforward certainly sounds enticing, especially given the anxieties many of us now feel faced with a growing ecological crisis, the resurgence of fascist authoritarianism and the grinding misery of inequality and injustice. But anyone who has ever attempted a posthuman research study knows just how quixotic and methodologically challenging it can be.

In an attempt to understand these tensions better ourselves, we here examine three interrelated methodological challenges facing posthuman researchers. The first concerns vitalism. Posthuman research looks for a much more inclusive, equitable distribution of life and creative vitality within the cosmos. In doing so, it has revived earlier forms of vitalism. Vitalism is a controversial concept, particularly among scientists and humanists, because it challenges conventional notions of causation, suggesting some ‘vital force’ lying behind events. The first methodological challenge for posthuman researchers, then, is to explain change and emergence. And as we will argue shortly, this has been done with varying degrees of success.

The second challenge concerns substance. With one or two exceptions, the entire history of philosophy has been underpinned by the belief that matter begins as fixed and stable and then is moved by some external force (see Nail, 2024 for a detailed analysis of this). From this idea we get the concept of being, identity, matter, objects and things, but also the belief in a super-natural, motive force lying *beyond* this world (Wallace, 2025). These transcendent forces could be in the form of the god(s) of the established religions, ancient spirit traditions, Platonic ideal forms, fate, the Freudian unconscious, mother nature or the universal laws of modern science. Posthuman research rejects these forms of transcendentalism however and looks instead for the immanent vitality in all things. The challenge for posthumanists here is to explain where life come from and why we have something and not nothing.

The third challenge is correlational. More than anything, posthuman research rejects the privileged human voice inherited from Kantian idealism. Immanuel Kant argued that we can never fully apprehend reality because our experience of the world is only ever available to us through our senses, and so ‘One cannot think or perceive something without thinking or perceiving it’ (Shaviro, 2014: 108–109). There is a central correlation between people’s thoughts and the objects *of* their thought. Posthumanism rejects this in principle but struggles with alternatives. How can we hear the more-than-human world without its voice sounding human?

These three challenges create significant methodological problems for posthuman researchers. Fortunately, over the last decade or more, there has been an enormous increase and interest in posthuman research, especially in healthcare, and a wide array of alternative approaches have been trialled. Some have been more successful than others, however. Our contention is that we can judge some of the quality of posthuman research based on the three challenges: vitalism, substance and correlation. We also contend that we should be able to use these three challenges to develop more coherent and compelling research in the future. This thinking runs alongside our own Posthuman Walking Project. Our goal in this study has been to develop an anti-correlational, immanent, process-based approach to the study of walking with long-term pain. After briefly outlining the project, we will unpack the three challenges in greater depth and show how existing approaches stand up against them. We close the article with some methodological recommendations and some meditations on our own experience of researching as posthumanists.

## The Posthuman Walking Project

This paper originated in a research collaboration between a group of critical physiotherapists, artists and persistent pain sufferers from the UK, Philippines, Norway, Canada and Aotearoa New Zealand, focusing on our shared interest in pain, walking, natureculture, and landscape. Walker-partners and researchers collaboratively developed ways of capturing and editing photographs, video and audio on mobile phones, alongside written or spoken commentary. Walker-partners and researchers worked together to develop ways of researching the environment as a force when walking with persistent pain. The Posthuman Walking Project (PWP) was funded by the Landscape Research Group in response to their call for research into landscapes of care.

An earlier related study used film and biomechanical data to explore people’s experiences of walking with persistent low-back pain (http://significantwalks.com). In the PWP we wanted to build on the experiential strengths of *Significant Walks*, but to foreground the non-human aspects of the environment across a range of social and cultural contexts.

Beyond the normal complexities of managing an international collaborative research study, we also faced some challenges particular to posthuman research: how might the environment be thought of as agential (whether a human experiences it as such or not); how might walking in pain traverse normal therapeutic boundaries; when and where do therapeutic occasions occur, begin and end; how can we represent human-environmental entanglements without being anthropocentric; and so on? There were questions of ecological inequities and concerns for the macro and micro-environments we were enmeshed in. And we were concerned with the ways we might encounter and capture more-than-human pain.

Our goals were twofold: to break away from the more familiar biomedical and human experiential healthcare research into pain, and to design the study consistent with posthuman theories and post-qualitative methodological principles. To that end, we:

1.  Critiqued any tendency to fall back into more familiar modes of inquiry, especially those that might emphasise systematic, linear, formulaic, normative or humanistic approaches
2.  Drew together our many and varied readings in posthuman and process philosophy, sharing thoughts on the ways we might use the writings of Barad, Bennett, Bergson, Braidotti, Deleuze, Guattari, Haraway, Nail, Whitehead and others to shape the study
3.  Remained open to the possibilities for any form of data capture that could de-emphasise human experiential judgement and foreground the more-than-human
4.  Developed our methodological approach iteratively, in constant dialogue between the researchers, walker-partners and, we hoped, the environments we participated in

Quantitative and qualitative research in pain has always been underpinned by a deep humanism, and we wanted to see if there was more to understanding pain than that which could be understood anthropocentrically. We were ‘fuelled by the active desire to actualize unprecedented modes of epistemic relations. . . acting on their supra-disciplinarity sensibility so that movement can be set in action towards a qualitatively new approach’ (Braidotti and Hlavajova, 2018: 45).

But, as many posthuman qualitative researchers have found, it is often easier to talk about composing a ‘meta-pattern indexed on the becoming-minoritarian of knowing subjects and knowledge practices’ (Braidotti and Hlavajova, 2018: 45), than it is to actually do it. And so, the question of how we could approach pain as a process of becoming, indeterminate and transversal: a process in which the human subjective experience and biological markers of pain represent only one (perhaps very small) part of pain occasions experienced by all things, came to be a familiar refrain throughout the study. What follows then is a condensation of some of the philosophical and methodological challenges and partial responses we generated in undertaking the project.

## A walk on the wild side

### Philosophies of affect, relation and entanglement

Without doubt, the most popular posthuman approaches in the healthcare literature to date have been approaches that we will call the philosophies of affect, relation and entanglement (PARE). These include critical/feminist new materialism (NM), actor network theory (ANT) and affect theories more generally (see Lupton, 2019 for summary of these approaches). NM – by far the most widely used PARE approach – seeks to remind us that life ‘is not the exclusive prerogative of humans’ (Braidotti, 2019), through methodologically diverse and inclusive, trans-disciplinary approaches, designed to surface a ‘realistic ethics attentive to the impact of human culture’ (Sheldon, 2015: 195), a caring concern for the ‘vivacity, vulnerability, and sometimes the surly intransigence’ (Sheldon, 2015: 195) of nature, and the non-discursive agency of other-than-human forces (Daigle and McDonald, 2023; Hird and Roberts, 2011). There are many branches of new materialism, but the most active – at least in healthcare – has been critical new materialism (CNM), which draws heavily on the works of writers like Alaimo, Barad, Bennett, Braidotti, Butler, Haraway and Irigaray, and challenges the structural, hierarchical ordering of different categories of human (Braidotti, 2013). The new materialisms draw philosophically from a loose assemblage of fields, including monism, vitalism and relational, multi-species intersectionality, but their epistemological focus is ‘emphatically relational’ (Sheldon, 2015: 195). Their ontology is underpinned by the non-discursive agency of other-than-human forces (Hird and Roberts, 2011), operating across what Fox and Alldred have called an ‘affect economy’ (Fox and Alldred, 2016).

New materialism is not without its critics, however and some of these criticisms have direct methodological bearing for anyone doing posthuman work. Object-Oriented Ontologist Graham Harman has suggested that all affect, relational and entanglement theories suffer from an over-reliance on relational agency which leaves them unable to explain creation and emergence. Because in PARE everything an entity is, or can become, is defined by its capacity to affect and be affected by another, all possible change must be contained *within* the relation. But where does ‘the new’ emerge from if there is no surplus, nothing ‘outside’ that might induce novelty? PARE cannot allow novelty to emerge from ‘outside’ because then affective relationships would not be enough. Which leaves PARE researchers with the problem of explaining how emergence and changes happen in the world, and whether relations are, indeed, sufficient.

A second problem facing PARE relates to the place of objects and things. When PARE researchers describe affective relations, they often assume the presence of ‘solid’ objects and things *in* relation. This is seen most starkly, perhaps, in the widespread use of Deleuze and Guattari’s assemblage theory, which often places Thing X in relation to Thing Y. This has resulted in a ‘tendency to treat the assemblage as a physical entity cobbled together from random bits of material like a potluck dinner or a patchwork quilt’ (Buchanan, 2020). All too often in new materialism, ‘people, hurricanes, and battles all get addressed in the same register’, and, in so doing, ‘it sanctifies a bloodless world by cataloguing the networks that make up its many attributes’ (Culp, 2016).

A third problem for PARE is anthropocentrism. PARE strives to be a *post*human philosophy, but it is hard to see sometimes how ‘post’ the human has become. Rosi Braidotti, for instance, criticised Actor Network Theorist Bruno Latour for offering a flattened ontology that ‘reject[s] the need for any theorization of [human] subjectivity’, thereby ‘undoing the possibility of a political project altogether’ (Braidotti, 2019). For Braidotti, it is human ‘Class, race, gender and sexual orientations, age and able-bodiedness’ of the many ‘missing people’ (Braidotti, 2019, emphasis added) that should be the focus for critical posthuman work. Braidotti, like many other posthumanists, is concerned to highlight ‘the evolving nature of the human subject and human constructed systems of power’ (Braidotti, 2019); a sentiment shared by many of the authors of new materialist work in healthcare in recent years (Cohn and Lynch, 2018; DeFalco, 2020; Dillard-Wright et al., 2024; Hodge and Olson, 2024; Malone and Tran, 2022; Renold and Ivinson, 2017; Richards, 2023; Sinclair and Mahboub, 2024). Jane Bennett has suggested that such anthropomorphism is both unavoidable (‘To put it bluntly, my conatus will not let me “horizontalize” the world completely’ (Bennett, 2009: 104)), and perhaps even desirable (‘Maybe it is worth running the risks associated with anthropomorphizing. . . because it, oddly enough, works against anthropocentrism’ (Bennett, 2009: 120)). But, as some people have asked, has this not simply revived a ‘completely anthropomorphic formulation’ (Nealon, 2021: 189); the very problem posthumanists were trying to avoid?

This is not a critique of the urgent necessity of critical emancipatory research, however. Rather it asks whether PARE is offering anything new. Terry Eagleton thinks not; ‘Like many an apparent innovation, New Materialism is by no means as new as it seems’ (Eagleton, 2017: 11). This has led authors like, Nealon, Buchanan, Lemke and others to argue that ‘it’s not critique that’s run out of steam’, but the ‘production of new meaning(s)’ (Nealon, 2021: 193). Perhaps ‘[t]he least-new thing about the new materialism is its founding commitment to making it new’ (Nealon, 2021: 89). Can other approaches be said to be any better in this regard, though?

### Speculative realism

In some ways, speculative realism (SR) does provide some novel formulations and gives researchers new insights and new tools, but in other respects it is quite conservative. SR came into being two decades ago in response to the ‘material turn’. From the outset, SR attempted to be anti-correlational. Correlationism was a term coined by Quentin Meillassoux (Meillassoux, 2008) to refer to the legacy of Kantian idealism. Where the natural sciences asserted that reality was mind-independent, Kant argued that we have no way of knowing whether a mind-independent reality exists, because everything we can know about the world is always filtered through our perceptions. Correlationism ‘disqualif[ies] the claim that it is possible to consider the realms of subjectivity and objectivity independent of one another’ (Meillassoux, 2008: 5). Consequently, we can never know the world *as such*, only as a correlation of thought. Speculative realism rejects the anthropocentrism of idealism and seeks to develop philosophies that accept the reality of the world as such *and* account for human and more-than-human experiences of it.

The most established and most widely cited approach to SR can be found in Graham Harman’s Object-Oriented Ontology (OOO). Like PARE, OOO is a relational ontology, but in OOO the relation is only superficial. Entities only ever encounter each other’s surfaces, and the glassy essence of the object always withdraws and exceeds any ability to be fully known (Harman, 2011, 2016). Harman draws heavily on Husserl and Heidegger to explain the fundamental withdrawal and excessive quality of things. OOO is avowedly posthuman, arguing for a flat ontology in which relations are equivalent regardless of form, human or otherwise (Morton, 2013, 2017).

Quentin Meillassoux has taken a different approach, arguing that the legacy of correlationism is that it ultimately favours thought and consigns being merely to ‘dumb’ matter (Meillassoux, 2008). Matter has no internal force for the correlationist; it is merely passive, awaiting the attention of the thinking human. So, if we are going to be able to engage as posthuman researchers, we need to understand a world that is ‘unaffected by whether or not anyone thinks it’ (Meillassoux, 2008: 116). To do this, we need to turn away from thought towards non-thought; towards a world with ‘no subjective-psychological, egoic, sensible or vital traits whatsoever’ (Meillassoux, 2012: 2). Meillassoux’s project, then, is to erase all subjectivity; to erase all sentience, perception and reason (Meillassoux, 2012: 6). To achieve this, Meillassoux turns to mathematics as a way to produce statements about the object ‘in itself’, anterior to consciousness (Meillassoux, 2008: 9).

A third approach comes from Ray Brassier. Brassier also turns to the sciences to rid ourselves of the psychological need for human-centred stories; to locate a world that is incomprehensible in narrative terms (Rychter, 2011). Brassier argues that we can use the physical sciences to measure the *inadequacy* of our conceptual understanding of things (Brassier, 2007). The result for Brassier is a form of positive nihilism; a nihilism borne of the belief that the logical conclusion of the hubristic human search for objective reality will ultimately result in the destruction of the manifest image of man (Brassier, 2007). But rather than seeing this as a message of despair, Brassier argues that if we can get past the idea of our own extinction, we might be better placed to understand the full extent of thought as it exists already, beyond the boundaries of our anthropocentrism.

What unites SR is its commitment to being anti-correlational, but results have been mixed. OOO especially has been criticised for paradoxically turning a principled desire to get away from Kantian Idealism into an ultimate form of subjectivism as the absence of correlation becomes its own object of thought (Shaviro, 2014: 122). Who is it, Carol Taylor asks, that renders the ‘alien’ object knowable? By whose criteria is this rendering deemed to be ’satisfactory’ (Taylor, 2016)? For many PARE writers, SR is evidence of a kind of ‘patrilineation’ occurring in posthuman research, that allows researchers to say nothing about the kinds of ongoing oppression that motivated posthumanism in the first place (Sheldon, 2015: 203; see also Lemke, 2014; Taylor, 2016: 210; Osborne and Rose, 2024). ’At the very moment when humans have caused a state shift in the earth’s biosphere and are presiding over a mass extinction’, Campbell et al argue, ‘we are witness to the ascendency of a social theory that massively redistributes agency to the nonhuman and promotes withdrawal as the primary mode of being’ (Campbell et al., 2019: 129–130). Not surprisingly, there is now a ‘particularly rancorous’ relationship between OOO and critical new materialists (Sheldon, 2015: 194).

Perhaps the most significant criticism of SR though is its reliance on a substance-based ontology. A substance-based ontology asserts that there is a mind-independent reality to the world. Forms, matter and objects exist whether we humans perceive them or not. The problem here is not the mind-independence of things, since the rejection of any human-centred meaning-making must be a corollary for any approach that claims to be truly posthuman. Rather, the problem is one of movement and transcendence. Substance-based ontologies are fundamentally static. Brute matter is lifeless, inert, and fundamentally passive; always relying on some agent or vital force beyond itself to animate it. In this respect, SR is deeply conservative, joining philosophers who have ‘dedicated their lives to the discovery of something genuinely immobile that could explain why things move’ (Nail, 2024: 2). To Aristotle’s unmoved mover, Descartes’s certain knowledge, Newton’s God as clockmaker, and even Einstein’s block universe, we can add religions and many of the world’s spirit traditions with their super-natural beliefs in God(s) and other ‘realms’ beyond the physical world, modern science’s belief in universal laws, psychology’s unconscious, even belief in luck, chance, fate and destiny. All of these assert, as SR does, that movement, flow, flux, becoming, change, creation and emergence are secondary properties of things.

If researchers are to be truly posthuman in their use of concepts like assemblage, becoming, conatus, creative evolution, duration, flux and flow, intuition, monads, nomadism and rhizomatics, their work cannot be substance-based. They cannot talk of matter and objects as if they are fixed or stable ‘things’, possessing some kind of mind-independent reality that requires an ‘other’ to bring it to life. In the most pragmatic sense, this means that researchers cannot fall back on any of the hallmarks of quantitative health research (positivism, classical empiricism, objectivity, variables, statistics, linear time, and so on). But neither can they draw from the language of identity and being, which puts most qualitative health research out of bounds. These heavily privilege Kantian idealism and correlate subjective experience with objective reality, which is deeply anthropocentric. Equally we cannot sidestep the problems of substance by focusing instead on pure relation as most PARE approaches do, since these approaches cannot explain change and causation without reference to some vital force *beyond* relation itself. Does a third approach – process philosophy – respond better to the problems of vitalism, substance and correlation, then, or is this also confounded by criticism and methodological confusion?

### Process philosophy

Although it has experienced something of a revival in recent years – particularly because of its affinity with quantum physics – process philosophy (PP) is, in fact, very old, with forms dating back to the pre-Socratic Greek philosophers, Daoism, Buddhism, some forms of early Hindu philosophy and a number of non-Western, non-theistic Indigenous cosmologies. The antecedents of contemporary forms of PP can be found in the works of Bergson, James, Leibniz, Nietzsche, Spinoza and Whitehead. And PP plays an important part in the work of writers like Bakhtin, deLanda, Deleuze, Kleinherenbrink, Lapoujade, Malabou, Manning, Massumi, Merleau-Ponty, Nail, Serres and Stengers.

Thomas Nail suggests that PP is first and foremost a philosophy of movement (Nail, 2024), opposed to the kinds of substance-based approaches that have dominated Western philosophy since the pre-Socratics; ‘Everything we thought was stable, from subatomic particles to the cosmos at large, has turned out to be in motion. . . The old paradigm of a static cosmos built from static particles is dead. All of nature is in perpetual ﬂux’ (Nail, 2021: 1). PP rejects the idea that matter is fundamentally fixed and static. The universe is not constituted by a cornucopia of ‘stuff’, and there is no abstract universal time against which these myriad ‘things’ move. There is only flow and the indeterminate ‘autonomous, ceaseless autoproduction of the real’ (Culp, 2016). Things that appear to be solid, bounded matter are, in reality, momentary thickenings and concrescences in the incessant flow of energy.

PP also rejects the idea that this endless flow depends on an external, transcendental, vital force to give it life. In PP, entities have everything they need to continue reproducing difference without reference to an external cosmic script. What drives this immanent process is an *anterior* force, an impulse, that is ‘so creative it belies any teleology’ (Kreps, 2015: 13). Several PP authors offer variations on this theme of onto-genesis. Bergson, for instance, spoke of creative evolution as a product of élan vital, which is the ‘undifferentiated unity existing prior to any individuation’ (Helin et al., 2014); the machine-like desire impelling the universe forwards. Nail favours dissipation and entropy. Whitehead calls this force the subjective aim. Deleuze prefers desire and Idea.

Whitehead suggests actual occasions (what in substance philosophy are called objects and matter) reach out, prehend, or feel into the world around them. But this is not a spatial palpation, but temporal. Time functions differently in PP. Time is neither universal nor distinct from world events. There is no aloof cosmic clock, ticking disinterestedly behind all things, but a ‘continuous progress of the past which gnaws into the future and which swells as it advances’ (Bergson, 1911: 4). The world of ‘things’ that we assume to encounter are merely ‘thickenings’ and folds, strata and molar aggregates in the ceaseless flows of duration. So, PP addresses the question of vitalism and the transcendentalism of substance-based approaches with immanence, élan vital, duration, movement, entropy and indeterminacy, and it addresses the correlational problem with pan-experientialism.

But to achieve this, PP creates several of its own problems. The first is with its language, which is notoriously opaque. Most process philosophers argue, for example, that Alfred North Whitehead’s 1929 masterpiece *Process and Reality* is an indispensable guide to posthuman process philosophy. But anyone who has tried to read it knows its reputation as one of the most difficult books in the canon is well earned. At times it is almost impenetrable. Deleuze and Guattari’s writings are no different. Here, for instance, is their methodological advice.

This is how it should be done: Lodge yourself on a stratum, experiment with the opportunities it offers, find an advantageous place on it, find potential movements of deterritorialisation, possible lines of flight, experience them, produce flow conjunctions here and there, try out continuums of intensities segment by segment, have a small plot of new land at all times (Deleuze and Guattari, 1987: 161).

Of course, the writings of these authors must have a degree of obscurity about them because they are trying to break free from the conventions of substance-based thinking. But this does not help the researcher trying to make sense of and use concepts like rhizomatics, prehension, intuition, the virtual, nomadism and concrescence in their work.

The second problem is somewhat related. Because PP is opposed to almost every facet of contemporary Western thought, from its belief in the nature of time and space, to the laws and super-natural forces that govern causality, to objecthood, identity and the existence of social structures, it repeatedly comes up against structural axioms and dogmatic images of thought that it has to overcome. For example, PP accounts for movement very well, but struggles more to account for materiality, just as substance-based approaches can account easily for objects, matter and being, but struggle to explain movement. Process philosophers often must pass *through* substance-based narratives first however, accounting for their ontological position through a substance-based philosophy lens. Quantum physicists face the same problem now, because they operate in an environment still governed by classical Newtonian thought. Unfortunately, in being forced to work this way, a lot of original ingenuity and creativity of process philosophy gets lost as researchers make pragmatic decisions about projects in order to fulfil the requirements of funding bodies and ethics committees and address the quizzical concerns of their fellow classically trained academics.

A third problem with PP – a problem shared with SR – is its quietism in the face of social and ecological injustice (Cooper, 2024; Lugt, 2025). By *de*-centring human power and influence, critics argue that posthumanism has supplanted a concern for identity and oppression, with performative and immanent modes of self-transformation (Coole and Frost, 2010: 9); a ‘bodacious’ and ‘excessive enchantment’ with things (Nealon, 2021: 69) which feeds directly into the rhetoric of a biopolitical neoliberalism (Nealon, 2021: 69; see also Brady, 2025). Posthumanism, Culp argues, is ‘a perfect fit in a world where capitalism produces subjectivity’ (Culp, 2016). PP and the broader posthumanisms, then, may be unintentionally performing ‘experiments in capitalist productivity’ (Beller, 2006: 27), effectively doing the wholly unintended work of ‘coding and commodifying new assemblages and new modes of enmeshment for capitalism’s gain’ (Braidotti, 2019).

## Methodological implications of process philosophy

Posthuman researchers from all three traditions – PARE, SR and PP – face some significant methodological challenges. The first concerns the way in which they explain emergence, causality and purpose in the universe, because they must do so without recourse to an ‘unmoved mover’. They must also do this within a system that absolutely abides by these classical axioms. Many posthumanists have turned to older forms of vitalism to help them explain emergence and the new. Some have looked to animistic and pre-modern, non-Western wisdom traditions. Others have turned to the arts, quantum physics, eliminativism, pan-psychism and non-scientific forms of expression to explore new horizons of thought. Often, though, these struggle to escape their latent anthropocentrism and their underlying concern for human accountability, deeper human understanding and, ultimately, human flourishing. So adopting an approach that can explain reality while also being non-vitalist, non-transcendent, and non-humanistic has proven difficult for many.

The second problem concerns correlation and the degree to which we humans are ever able to know the world *as such*, and not merely as a projection of our own subjective experience and human cognition. There is clearly a great deal of life going on in the cosmos that ‘we’ have little awareness of or direct involvement in. But how can we know this without, at the same time, turning our research into the product of our own imagination? Posthuman researchers are concerned with the vast more-than-human world. But is every attempt to be more-than-human hamstrung by our subjective perspective? Can we ever truly rid ourselves of our subjectivity? And if so, should we? If we cannot, does posthumanism really offer us anything new; anything that hasn’t already been addressed by historical materialism and critical theory? Some have suggested eliminativism as a strategy (especially in SR), but others suggest that this is just an apology for the perpetuation of patriarchal objectivity, neoliberal biopolitics and the abandonment of social advocacy.

The third problem is how to account for movement and ‘the real’. If we embrace, as many posthuman researchers appear to do, concepts drawn from process philosophies (rhizomes, becoming, flux and flow, indeterminacy, and so on), we cannot at the same time fall back into the language of things, matter, objects, being and identity. There is no ‘is’ in process philosophy. How then should posthuman research proceed, given these challenges and difficulties?

We suggest four steps: attention to key principles, concept creation, deep reading and paying attention to slippage.

Firstly, attention to key principles. In Table 1 we have attempted to define seven principles to underpin any posthuman methodological endeavour. These are pan-experientialism, creation, indeterminacy, process, movement, immanence and duration. We suggest any research study that claims to be posthuman should be assessed against these principles, and any methodology must reflect them.

**Table 1.** Guiding principles for posthuman process-based research.

Promote	Avoid
Pan-experientialism: Thought, feeling, experience, self-expression and self-actualisation involving all processes at all scales	Humanism/correlationism: Life as seen through human senses, humans as apex social actors, universal purpose bent towards human flourishing
Creation: Life is genetic, creative evolution is an unrelenting experiment in the creation of the new	Discovery: Life is a pattern, a mystery to be solved through rigorous investigation
Indeterminate: The creation of newness without defined limits. Not ‘absence’ but not distinct presence either. A process of becoming.	Determinate: Being, defined, limited presence.
Process: Reality is in constant motion, flux, never-ending flow, and becoming	Substance: Matter, things, objects, being and fixed identities are building blocks of reality
Movement: Life is always moving, change demands interaction but flows are already alive	Stasis: Life begins as inanimate static, fixed matter and is enlivened through interaction with other matter
Immanence: Everything a process needs is fully present	Transcendence: Life relies on another ‘realm’, an unmoved mover, to enliven matter
Duration: Duration is non-linear, rhizomatic and anarchic, the past folds into the present and the future is radically empty	Time: The arrow of time is linear and measurable and runs from the past to the future

Although no formalised posthuman methodological schema could ever exist, we have drawn from some recent works that have tried to frame posthuman inquiry. These include David Lapoujade’s aberrant movements (Lapoujade, 2017), Arjen Kleinherenbrink’s machine ontology (Kleinherenbrink, 2018), Thomas Nail’s philosophy of movement (Nail, 2024), Alfred North Whitehead’s philosophy of organism (Emmet, 2021), and Deleuze and Guattari’s schizoanalytic general schema (Deleuze and Guattari, 1987). Charles Hartshorne, Erin Manning and Jenny Helin et al have also offered more granular methodological guidelines (Hartshorne, 1979; Helin et al., 2014; Manning, 2016).

Deleuze reminds us that our goal should always be to practice a ‘minor science’ (Deleuze and Guattari, 1986); one that is nomadic, eccentric, generative and organic, in contrast with the kinds of ‘Royal’ science that we are all so familiar with in healthcare research. The challenge is to ‘denaturalise’ (St. Pierre, 2017) the dogmatic image of thought common to health research.

The second step must be the creation of concepts. Posthuman philosophy is first and foremost a philosophy of concept creation rather than discovery. Often, ‘[T]he solution of problems is trivial compared to their *stating*’ (Brady, 2022), and so the work of the posthuman philosopher centres on their ability to invent and posit problems, not uncover them (Brady, 2022);

We are socialised to see truth as solutions. This begins in childhood when the teacher poses all of the problems and the pupil’s task is to derive the best fit for the truth the teacher desires. For Deleuze this is a kind of slavery, but very much followed through in institutions like training and the professions (Shaviro, 2010).

But as Deleuze, Whitehead and Nietzsche showed, solutions come wrapped within the problems they define. So, although they may be obscured, a ‘speculative problem is solved as soon as it is properly stated’ (Deleuze, 1988: 15). The task is not to uncover the solution, but to properly define the problem and, in so doing, ‘creat[e] the terms in which it will be stated’ (Deleuze, 1988: 15). In posthuman inquiry, finding and positing problems is far more important than solving them; ‘Philosophical concepts are not for all time; they are not given in advance’ (Shaviro, 2010); they ‘are not waiting for us ready-made, like heavenly bodies’. Instead, they must always be ‘invented, fabricated, or rather created afresh’ (Shaviro, 2010).

Deleuze and Guattari refer to concepts as territories of thought, open-ended constellations of virtual singularities which can be extracted as an event from the flow of thinking. To create a concept is to make a thought-event reproducible for other thinkers – to give thinking new places to go*’*(Cordry, 2025).

In posthumanism, every occasion of emergence, change, and the new is an act of concept creation. All movement, flux and flow is only the creation of new problems, new concepts. Slime moulds do this, the wind does it, love does it, movement itself does it. Concept creation, then, is ontogenetic; it enacts difference, feeding the creation of the new; continually improvising and experimenting. New concepts call forth ‘forces in thought that are not the forces of recognition, today or tomorrow, but the powers of a completely other model, from an unrecognized and unrecognizable terra incognita’ (Deleuze, 1993: 136). Our work as posthuman researchers, then, is less about digging for buried treasure, and more about plugging in to the movement as it happens; trying to write the problem as we go.

Which brings us to the third key principle, deep reading. In trying to devise his own method of movement, Deleuze was drawn to artists and thinkers who broke convention and created new ways of thinking and practicing: avant garde writers like Carroll, Joyce, Proust and Woolf, painters like Francis Bacon, poets and performance artists like Antonin Artaud. But he was also a thoughtful student of mainstream ideas. He wrote books on Bacon, Bergson, Foucault, Kant, Sacher-Masoch and Nietzsche, and Proust, and wrote deeply about Hume, logic and empiricism, Spinoza and Lacan. A few years ago, Mark Tesar quipped that post-qualitative researcher Bettie St. Pierre had ‘said to me once that she can always tell who has been reading, and who has not!! That sentence frightened me to death, but I do agree that reading and thinking with posthumanism is a more complex process than some scholars or research students actually realize’ (Tesar et al., 2020: 138).

This demand for deep reading creates some serious difficulties for posthuman healthcare researchers, especially if they are trained as clinicians not philosophers. How far should one go to truly understand posthumanism? Is it enough to read Bergson, Deleuze, Foucault, Nietzsche and Whitehead from introductions and secondary sources, or should you read only the primary texts? Should you read Leibniz, Hume, Spinoza, Schopenhauer and all of the other philosophers who shaped the ideas of these posthumanists? Should you learn a new language in order to read the works in the original? How far should you go in order to know whether you have indeed created a new problem, a new concept? Perhaps everything that you are thinking about has been thought about before and is perfectly captured in concepts like aberrant movement, agencement, anarchiving, actual occasions, body-without-organs, concrescence, creative evolution, desiring production, dissipation, eternal objects, eternal recurrence, haeccity, intensity, multinaturalism, nomadism, pink noise, pre-acceleration, supernormal, prehension, umwelt or other concepts found in posthuman theorising today.

The other reason for this deep reading relates to our fourth key principle: attention to slippage. Because posthumanism – especially that branch based in process philosophy – runs counter to most of the normal practices of healthcare research, it is crucial that we recognise when there might have been some ontological slippage. This is particularly common when our work betrays an underlying substance-based approach or focuses too heavily on questions of human flourishing at the expense of the more-than-human. Paying attention to latent vitalism, substance and correlation gives posthuman research its rigour but, more than this, it keeps posthuman research moving and prevents it from becoming fixed and immobile: a ‘thing’.

## Discussion

Thinking again about our Posthuman Walking Project, we have encountered problems that are perhaps shared by all posthuman researchers. The first has been the challenge to remain true to posthuman philosophy. We have spent hours discussing our tendency to want to discover rather than create; to measure things and thereby spatialise objects and give them solid form. We have used hyphenated assemblages of things as shorthand, when we ought not to. We have slipped into notions of the uniform, immutable ‘self’. And we have brought causal social structures into our discussions and betrayed our personal histories in advocacy and activism.

We have also struggled mightily with the theory. None of us is a trained philosopher and we have all felt dilettantes at times. Posthumanism is such a vibrant area, which is enormously stimulating in some ways, but there are so many conflicting voices to take in – all with their own influences and antecedents – that it can feel overwhelming at times. And there are many overlaps and subtle variations to the different theories, that it can take months or even years, to tease apart all of the nuances. None of the philosophers we have drawn on as part of the PWP speak explicitly about pain or walking, which has caused us to ponder whether these events even have a place in posthuman philosophy; whether there are parallel concepts that might be adequate substitutes for pain and walking; or whether the creation of new concepts might even be possible.

Perhaps most of all, we have grappled with how to ‘do’ a posthuman, process-focused, post-qualitative study. There is no shortage of fine verbiage explaining the principles of immaterial becoming, affective assemblages, processes of concrescence and deterritorialisation but, to date, there have been very few researchers who have been successful in putting these principles into practice, especially in healthcare. This is perhaps understandable, because so much healthcare research currently operates within a substance-based system.

In this paper, then, we have tried to examine how it might be possible to construct a compelling methodological approach true to posthumanism in the face of myriad challenges. Our contention has been that while there have been many expressions of posthuman ideals in recent years, there remains a lack of clarity on how a researcher might proceed. We have argued here that all posthuman approaches have their strengths and limitations, but, for us, a process-based approach found in the works of philosophers like Bergson, Deleuze, Nail and Whitehead comes closest to the heart of posthumanism. Setting out these tenets, we have tried to show how a focus on key principles, concept creation, deep reading, and attention to ontological slippage might form the starting point for posthuman research to come.
